# Is there a link between diabetic glomerular injury and crescent formation? A case report and literature review

**DOI:** 10.1186/1746-1596-7-46

**Published:** 2012-04-20

**Authors:** Naoko Otani, Tetsu Akimoto, Wako Yumura, Daisuke Matsubara, Yoshitaka Iwazu, Akihiko Numata, Takuya Miki, Fumi Takemoto, Noriyoshi Fukushima, Shigeaki Muto, Eiji Kusano

**Affiliations:** 1 Division of Nephrology, Department of Internal Medicine, Jichi Medical University, 3311-1 Yakushiji, Shimotsuke-Shi, TOCHIGI, 329-0498, JAPAN; 2 Department of Integrative Pathology, Jichi Medical University, 3311-1 Yakushiji, Shimotsuke-Shi, TOCHIGI, 329-0498, JAPAN

**Keywords:** Diabetes, Diabetic glomerulosclerosis, Glomerular crescent, Hyalinosis, Exudative lesion, Renal biopsy

## Abstract

Glomerular crescents are most commonly associated with rapidly progressive crescentic glomerulonephritis; however, they also develop in response to a wide range of primary and secondary glomerular injuries. Since various kind of glomerulopathies occasionally overlay diabetic glomerular injuries, the presence of crescents in renal biopsy specimens of diabetics may have stimulated a search for etiologies other than diabetes. In this report, we describe an unusual case of diabetic glomerulosclerosis with peculiar extracapillary proliferation. Although such a relationship has so far been ignored in most of the literature, the etiological linkage between diabetic glomerulosclerosis and the development of crescents may not be exceptional. We have reviewed the previous literature and herein discuss the pathological implications of the development of crescents in patients with diabetic glomerulosclerosis.

**Virtual slides:**

The virtual slide(s) for this article can be found here: http://www.diagnosticpathology.diagnomx.eu/vs/3950457896920255.

## Introduction

Glomerular crescents, which are formed by the accumulation of cells in Bowman`s space that surround and compress the glomerulus, are most commonly associated with rapidly progressive crescentic glomerulonephritis (RPGN); however, they also develop in response to a wide range of primary and secondary glomerular injuries, including lupus nephritis, purpura nephritis, IgA nephropathy, post-infectious glomerulonephritis, and membranoproliferative glomerulonephritis (MPGN) [1-3]. Not surprisingly, these glomerulopathies occasionally overlay diabetic glomerular injuries [4-7]. Therefore, the presence of crescents in renal biopsy specimens of diabetics may have served to stimulate a search for etiologies other than diabetes [8]. In this report, we describe an unusual case of diabetic glomerulosclerosis with peculiar extracapillary proliferation.

## Case presentation

A 53-year-old male was admitted to our hospital in July 2010 with chief complaints of progressive swelling of the legs and shortness of breath. He had gained approximately 25 kg in the past five months. Eleven years prior to admission, he was found to have type 2 diabetes with a fasting blood glucose (FBS) of 200 mg/dl and HbA1c of 8.2, for which he had received sporadic medical care. However, he gradually developed a loss of appetite for psychosomatic reasons, making the use of hyperglycemia controlling agents unnecessary in the beginning of January 2010 (HbA1c 4.9). He neither smoked nor drank alcohol and denied using any drugs. No apparent past history of any renal disease was noted.

A physical examination completed on admission revealed that the patient’s face was swollen and edema was noted in the upper and lower extremities. The patient’s blood pressure was 224/140 mmHg, his pulse was 78 beats/min, his respiratory rate was 12 breaths/min, and his temperature was 35.2°C. Although the patient’s oxygen saturation was 97 percent while he breathed ambient air, a chest X-ray demonstrated the presence of bilateral pleural effusion. There was no rash or lymphadenopathy, and no petechiae were found. The patient’s heart sounds were normal. Renal sonography revealed that the size of both kidneys and each renal cortex echogenicity were normal, while a cardiac echogram indicated a minimal amount of pericardial effusion with normal left ventricular contraction. The laboratory data obtained on admission are summarized in Table 1. Tests for the presence of anti-neutrophil cytoplasmic antibodies (ANCA), anti-glomerular basement membrane (GBM) antibodies, anti-nuclear antibodies, hepatitis B virus surface antigens (HBsAg), anti-HBsAg antibodies, and hepatitis C virus antibodies were all negative. No elevations in either antistreptolysin or antistreptokinase were found. The patient’s urine contained 4.7 g of protein in a 24 hour specimen and was found to be 3+ for protein and 2+ for occult blood. The proteinuria selectivity index was 0.426 and creatinine clearance was 27.7 ml/min. The sediment contained 43 to 44 granular casts per low power field and three to four red blood cells per high power field. The urinary excretion of β2-microglobulin and N-acetyl-beta-D-glucosaminidase were 1061 μg/l and 64.6 U/g·Cr, respectively. Serum and urinary protein electrophoreses showed no increase in the level of monoclonal protein. An ophthalmologic analysis revealed the patient to have proliferative diabetic retinopathy.

**Table 1:** Laboratory data on admission

**White blood cell**	**7500/μl**	**C-reactive protein**	**0.38 mg/dl**
Hemoglobin	10.0 g/dl		
Platelet count	31.5 × 10^4^/μl	IgG	1211 mg/dl
		IgA	583 mg/dl
Blood urea nitrogen	26 mg/dl	IgM	153 mg/dl
Serum creatinine	1.37 mg/dl		
Total protein	4.9 g/dl	C3	108 mg/dl
Serum albumin	1.0 g/dl	C4	34 mg/dl
Sodium	146 mmol/l		
Potassium	4.1 mmol/l	FBS	97 mg/dl
Chloride	115 mmol/l	HbA1c	5.00%
Calcium	7.1 mg/dl		
Phosphorus	3.9 mg/dl		
Asparate aminotransferase	16 U/l		
Alanine aminotransferase	7 U/l		

**Figure 1:**
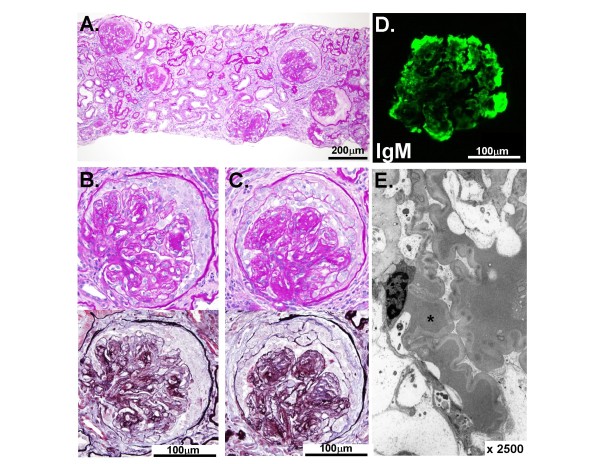
**Photomicrographs of the renal biopsy specimen.** ( **A**) A low power view showing the diffuse distribution of sclerotic glomeruli with or without various stages of crescents. ( **B and C**) Two sections of the same glomerulus with a cellular crescent. (Upper panel, *Periodic acid-Schiff stain*; Lower panel, *Silver Methenamine-Masson trichrome stain*) ( **D**) Immunohistochemical staining with an anti-serum specific for IgM showed focal entrapment in the depending portions of the areas of hyalinosis. ( **E**) An electron micrograph showing a portion of the glomerulus indicating an amorphous electron-dense material presumably representing hyalinosis or nonimmunologic deposition of plasma proteins (*). The scale or scale bar is indicated in each panel.

A renal biopsy was performed in the beginning of August (Figure 1). In total, two biopsy cores were obtained and observed carefully to identify the presence of the renal cortex and to guide sectioning of the material. Each core was divided so that the tips were immediately fixed with 2.5% glutaraldehyde for electron microscopy. One half of each remaining core was fixed with phosphate-buffered 4% paraformaldehyde and then was evaluated by light microscopy with standard pathological staining, including hematoxylin-eosin, periodic acid-Schiff, and silver methenamine-Masson trichrome stains, and the other half was frozen in liquid nitrogen for an immunofluorescence analysis. The specimens were stored at 4°C (samples for electron and light microscopy) or −80°C (samples for frozen sectioning) until final processing. Under a light microscope, 17 glomeruli, four of which were globally sclerotic, were identified within the renal parenchyma. Although hyalinosis lesions were seen in several glomeruli, there was no evidence of either vasculitis or fibrinoid necrosis in the blood vessels. Global widening of the glomerular mesangial regions and a number of rounded acellular mesangial nodules were identified in the rest of the glomeruli. The presence of fibrous and cellular crescents, without focal segmental necrotizing lesions, were confirmed in seven and two glomeruli, respectively. Congo red staining was negative. Electron microscopy revealed glomerular collapse with wrinkling of the basal lamina, foot process effacement of the glomerular podocytes, and amorphous electron-dense material in a capillary loop and the mesangial matrix. Immunofluorescence staining failed to demonstrate the linear staining of IgG along the glomerular capillary wall. Instead, the presence of focal granular depositions of IgM and C3 in the depending portions of the areas of hyalinosis were confirmed.

We presumptively diagnosed the patient as having advanced diabetic glomerulosclerosis with peculiar extracapillary proliferation. Thereafter, treatment with oral olmesartan medoxomil at 5 mg/day, controlled-release nifedipine at 60 mg/day, and furosemide at 80 mg/day was commenced. The patient also received psychosomatic care for anorexia. Although the patient’s periorbital, arm, and leg edema disappeared over a one month period, his glycemic control worsened with the improvement of his appetite, thus necessitating the use of hypoglycemic agents, including insulin. The patient’s urine protein settled around 1.5 g/g·Cr to 2.0 g/ g·Cr with a slightly elevated serum level of creatinine of 1.7 to 1.9 mg/dl at follow up without any exacerbation of the blood pressure control.

## Discussion

Nodular glomerulosclerosis, which is one of the major pathological findings of diabetic glomerulosclerosis, is a glomerular change characterized by nodular mesangial sclerosis and accentuated glomerular lobularity [9,10]. Numerous glomerulopathies resemble these morphological alterations when viewed under light microscopy: hypertensive renal disease, light chain deposition induced by a plasma cell dyscrasia, MPGN, and occasionally, amyloidosis [11]. In our case, no etiological linkages between these diseases and the glomerular injuries, except that between hypertension and glomerular damage, were confirmed. Indeed, two glomeruli that were detected on low magnification of renal biopsy specimens obtained from the patient showed collapsed glomerular tufts with wrinkling of the capillary walls and urinary space filled with acellular material, which seemed to be compatible with the features of ischemic glomeruli in hypertensive patients [12].

Apart from the diagnosis of type 2 diabetes eleven years prior to admission and the results of the ophthalmologic analysis, one may argue that there may have been no clear clinical evidence of diabetes in this patient, since normal values of FBS and HbA1c were noted on admission. However, a transient improvement in glucose metabolism is not exceptional in diabetics with eating disorders [13]. This was the case with this patient, as psychosomatic care for anorexia initiated after the renal biopsy resulted in the gradual deterioration of the patient’s glycemic control, thus necessitating the administration of insulin. The most characteristic immunofluorescent change in diabetic glomerulosclerosis is shown in diffuse linear staining of the glomerular and tubular basement membranes, although this is not necessarily true for all cases with diabetic glomerulopathy and the intensity of such staining varies between individual diabetics. Hyalinosis or exudative lesions often stain brightly with IgM and C3, as demonstrated in our patient [7,14,15]. Although the precise process underlying the development of such lesions remains to be delineated, it has been suggested that the development of lesions is associated with endothelial injury and possible hemodynamic alterations [16]. Consequently, it is reasonable to consider that the patient’s low compliance with the overall management of his diabetes and associated complications, such as hypertension, for the long time period prior to his admission may have played a pivotal role in the acceleration of microvascular and endothelial injuries, thus resulting in the various degrees of sclerotic changes compatible with diabetic glomerulosclerosis.

The presence of glomerular electron-dense material and the absence of various serological disorders associated with the development of crescentic glomerulonephritis may indicate the contribution of latent immune-based processes to the development of crescentic glomerular injuries in this patient [2]. However, the hyalinosis or exudative lesions seen in diabetic glomerulosclerosis are also known to be part of an accumulation of homogenous electron dense material [14]. Therefore, it may be difficult to distinguish non-immune hyaline accumulations from granular, electron-dense immune deposits [14]. Nevertheless, the correlations with the clinical, laboratory, and light and immunofluorescence microscopic findings in the current patient do not seem to support the possibility of a superimposed immune-mediated glomerular injury. Instead, the fact that there were varying stages of crescents with exudative lesions compatible with diabetic glomerular injuries shown in the renal biopsy of this patient finally led us to consider that the diabetic glomerular damage could be linked to the formation of crescents, which has been mentioned by a previous study [8].

In diabetics with overt proteinuria, a diagnosis of diabetic kidney disease can be made in the appropriate clinical setting without pathological confirmation in cases with diabetic retinopathy, a long duration of diabetes, and hypertension [7]. On the other hand, performing a renal biopsy on diabetics has usually been considered when the presence of renal disease other than diabetic kidney disease is suggested by clinical signs, such as the rapid deterioration of renal function, the detection of microscopic or macroscopic hematuria, or the presence of proteinuria in newly diagnosed diabetics without any retinopathy or neuropathy [6,7]. In this context, the information available concerning the qualitative and quantitative renal morphology of diabetic glomerular injuries seem to involve an intrinsic selection bias [6,7,17], and the presence of crescents within the glomeruli damaged by diabetes may therefore sometimes be overlooked especially in the diabetics with potentially advanced diabetic glomerular injuries as demonstrated in the current report. Subsequently, it is not surprising that the etiological linkage between diabetic glomerulosclerosis and the development of crescents has been barely mentioned in previous literature. Nevertheless, such a relationship may not be exceptional [2,8,18]. Apparently, the diagnostic and clinical impact of glomerular crescents in patients with diabetic glomerulosclerosis should therefore be evaluated more carefully.

## Conclusion

We herein described an unusual case of diabetic glomerulosclerosis with peculiar extracapillary proliferation. Although the etiological linkage between diabetic glomerulosclerosis and the development of crescents has so far only barely been mentioned in previous literature, such a relationship may not be exceptional. Further studies and accumulated experience with renal biopsies are required to better determine the diagnostic and clinical impact of glomerular crescents in patients with diabetic glomerulosclerosis.

## Consent

Written informed consent was obtained from the patient to publish this case report and any accompanying images. A copy of the written consent is available for review by the Editor-in-Chief of this journal.

## Competing interests

The authors declare that they have no competing interests.

## Authors’ contributions

NO and TA drafted the manuscript. YI, TM, AN, and FT made contributions for the acquisition of the clinical data. DM and NF carried out the tissue staining, immunoassays, and electron microscopic examination. TA, WY, NF, SM and EK conducted the analysis of the histological features and clinicopathological relations. All authors have read and approved the final manuscript.
